# FANCI: from genome guardian to translational therapeutic target

**DOI:** 10.3389/fonc.2026.1885055

**Published:** 2026-06-29

**Authors:** Xiaoyue Zhang, Yang Zhang, Mengying Chen, Jinmei Wei, Yanling Li, Tao Dai, Junyu He, Chen Han, Yanhong Zhou

**Affiliations:** 1 Department of Radiation Oncology, The Affiliated Cancer Hospital of Xiangya School of Medicine, Central South University/Hunan Cancer Hospital, Changsha, Hunan, China; 2 Cancer Research Institute, Basic School of Medicine, Central South University, Changsha, Hunan, China; 3 Medical Record Management and Information Statistics Center, Xiangya Hospital, Central South University, Changsha, Hunan, China; 4 Department of Nuclear Medicine, The Affiliated Cancer Hospital of Xiangya School of Medicine, Central South University/Hunan Cancer Hospital, Changsha, Hunan, China; 5 Urology Department, The Affiliated Cancer Hospital of Xiangya School of Medicine Central South University/Hunan Cancer Hospital, Changsha, Hunan, China; 6 Department of Clinical Laboratory, The Second People's Hospital of Hunan Province (Brain Hospital of Hunan Province), Changsha, Hunan, China

**Keywords:** DNA damage repair, FANCI, interstrand crosslink (ICL), malignant tumors, targeted therapy

## Abstract

Fanconi anemia complement group I (FANCI), a core component of the Fanconi anemia pathway, has emerged as a potential oncogenic factor across multiple cancer types. Located on chromosome 15q26.1, FANCI encodes a protein named FANCI that forms a stable heterodimer with FANCD2, playing a central role in the DNA damage response and repair. Beyond its involvement in DNA repair, FANCI participates in ribosome biogenesis, meiosis, and mRNA export, underscoring its essential role in maintaining genomic stability. Although FANCI deficiency has traditionally been associated with Fanconi anemia, recent studies have demonstrated that FANCI is overexpressed in various malignancies, where it promotes tumor progression and correlates with poor prognosis. The expression and activity of FANCI are tightly regulated at multiple levels, including transcriptional and post-transcriptional regulation by transcription factors and non-coding RNAs, as well as post-translational modifications such as phosphorylation mediated by PP2A and ATR, monoubiquitination catalyzed by the FANCL–UBE2T complex, and deubiquitination by USP1-UAF1. This review summarizes the functional mechanisms of FANCI across diverse physiological and pathological processes, highlighting its role as a crucial molecular bridge linking genomic stability maintenance to cancer development, and emphasizing its potential as a promising therapeutic target in cancer.

## Graphical Abstract

**Figure:**
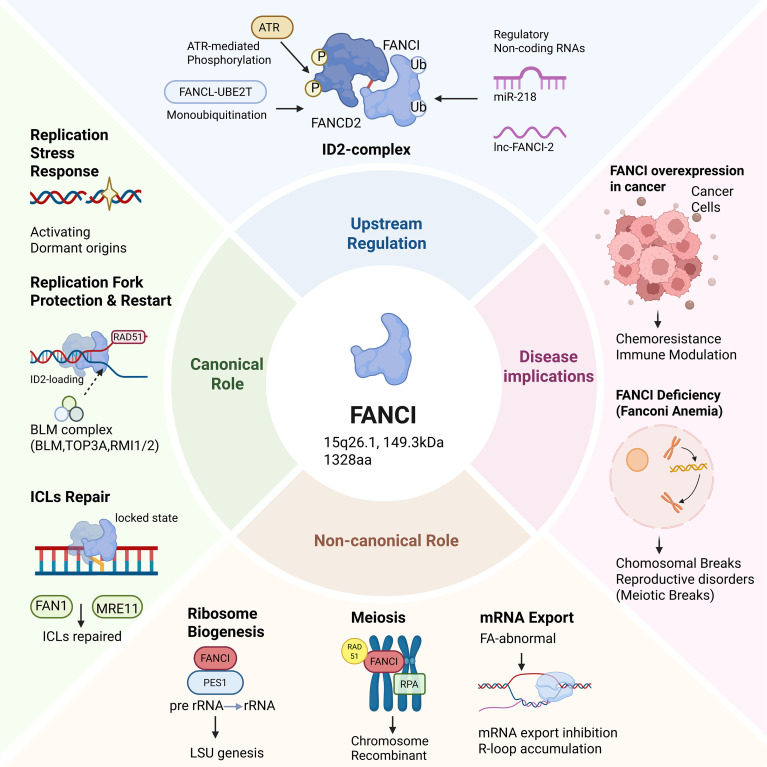

## 1 Introduction

Genomic stability is essential for cellular homeostasis, with DNA damage repair serving as a key mechanism to prevent the lesion accumulation from stress (1). While defective repair drives disease progression, enhanced repair can promote tumor survival and therapy resistance (2). Fanconi Anemia Complementation Group I (FANCI), also known as KIAA1794 or FAP250, is a core component of the Fanconi anemia (FA) pathway. The gene is located on chromosome 15q26.1, spans approximately 73.3 kb, and is highly conserved (3, 4). It encodes the FANCI protein, which comprises 1,328 amino acids and localizes to both the nucleus and cytoplasm. The pivotal role of FANCI in DNA damage repair is largely attributable to its ability to form a heterodimeric FANCI-FANCD2 complex (ID2), which features a DNA-encircling central cavity and a protruding tower-like domain (5, 6). Early structural studies showed that the ID2 complex adopts a clamp-like architecture encircling DNA (7). while more recent work demonstrates that it dynamically scans DNA and preferentially recognizes double- to single-stranded junctions, thereby functioning as a key DNA damage sensor (8).

As an essential component of the FA pathway, FANCI has traditionally been studied in the context of Fanconi anemia, a hereditary disorder characterized by bone marrow failure (9–12). However, accumulating studies have revealed that FANCI plays a broader role in cancer biology (13). Aberrant expression of FANCI has been observed across multiple malignancies, where it enhances DNA repair capacity, promotes replication stress tolerance, and supports tumor cell survival under adverse conditions (14–21). Beyond its canonical function in DNA repair, FANCI has been implicated in additional processes such as ribosome biogenesis (22), R-loop regulation (23, 24), and meiosis (25). Despite this expanding recognition, the molecular mechanisms underlying these diverse roles remain incompletely understood. This review aims to systematically consolidate current knowledge of FANCI’s functions across both physiological and pathological contexts, with a particular focus on evaluating its potential as a target for clinical translation.

## 2 Structural and functional characteristics of FANCI protein

FANCI is a versatile DNA-binding protein capable of recognizing a broad spectrum of DNA structures, including single-stranded DNA (ssDNA), double-stranded DNA (dsDNA), and various branched forms. The primary DNA-binding region of FANCI has been mapped to amino acids 200–1000 (8). Structural studies within this region have identified an ARM repeat domain spanning residues 985–1207. This domain contains a helix-turn-helix (HTH) motif that mediates direct contact with dsDNA. Moreover, the ARM repeat domain exhibits an intrinsic propensity to oligomerize, adopting monomeric, dimeric, and higher-order oligomeric states. This structural plasticity is believed to enable FANCI to adapt to and process diverse DNA substrates during interstrand crosslink (ICL) repair (26).

**Figure 1:**
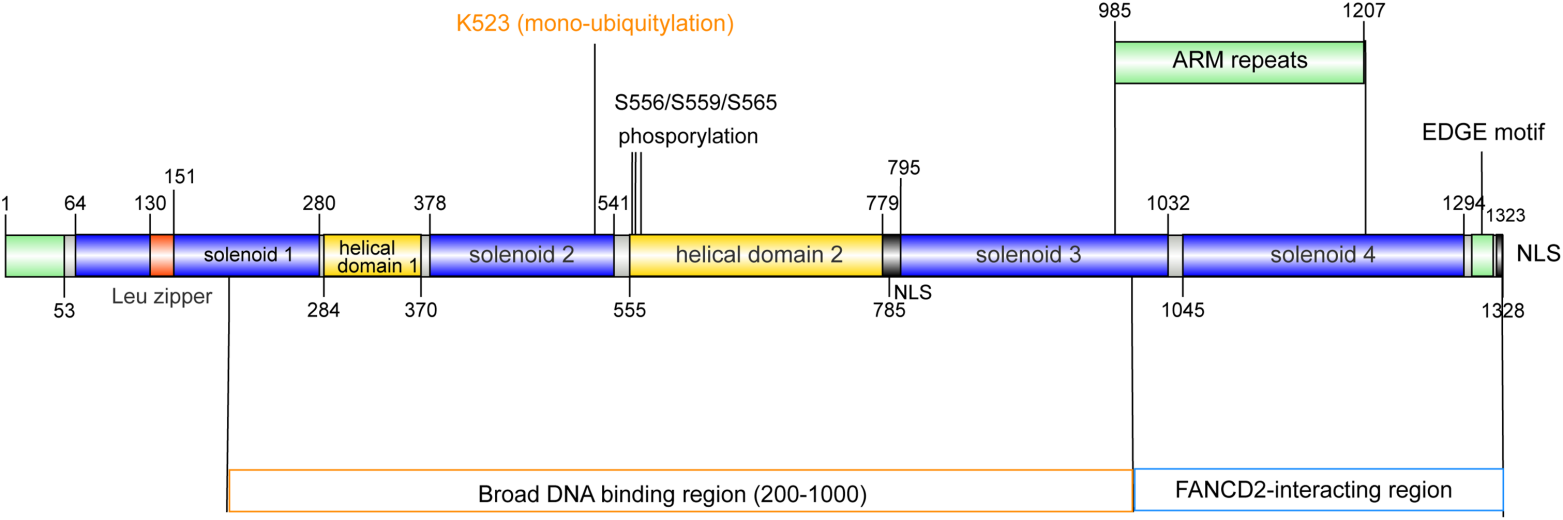
Structural features of FANCI.

Beyond its role as a broad-spectrum DNA structure recognizer, FANCI serves as a key regulator of the nuclear localization of the ID2 complex. Its nuclear import is mediated by two nuclear localization signals (NLSs) within FANCI (residues 779–795 and 1323–1328) (8), along with an NLS located in the N-terminal 58 amino acids of FANCD2 (27). Deletion of these NLSs results in the cytoplasmic mislocalization of FANCI, ultimately abrogating the DNA damage response (28).The C-terminal EDGE motif, while dispensable for ID2 monoubiquitination and foci formation, is essential for efficient ICL repair (5). It constrains FANCI’s non-specific DNA binding to fine-tune ID2 complex substrate selectivity during ICL repair, and may also promote timely ID2 dissociation from repaired DNA or recruit downstream repair factors (5, 12) (**Figure 1**).

Collectively, the multifaceted functions of FANCI—including broad-spectrum DNA recognition, nuclear import, and partnership with FANCD2—are coordinated through its distinct structural domains. In contrast, the precise function of the C-terminal EDGE motif and its molecular role in ICL repair remain enigmatic, necessitating further studies.

This schematic shows the domain architecture of the 1328-amino-acid FANCI protein. Key color-coded features: blue (solenoid domains 1–4), yellow (helical domains 1–2), green (solenoid 1 cap), red (leucine zipper), black (two NLSs). Indicated elements include functional domains/motifs (ARM repeat: 985–1207; EDGE motif: 1300–1303), critical modification sites (monoubiquitination: K523; phosphorylation: S556/559/565), and major functional regions (DNA-binding: 200–1000; FANCD2 interaction: 1000–1328) [Created by IBS 2.0 (29)].

## 3 FANCI participates in DNA replication and damage repair

FANCI safeguards genome integrity by orchestrating dormant replication origin activation, stalled replication fork protection/restart, ICL repair, and cell fate regulation in response to irreparable damage, underscoring its central role in maintaining genomic stability.

### 3.1 FANCI regulates DNA dormant replication origin activation

In mammalian cells, the initiation of DNA replication is tightly controlled through two cell cycle-dependent steps: the licensing of replication origins during the G1 phase and their subsequent activation in the S phase (30). When replication forks stall due to stress, cells activate licensed dormant origins to ensure complete genome duplication (31). Research indicates that under conditions of mild replication stress, non-phosphorylated FANCI is recruited to dormant origins. There, it interacts with the minichromosome maintenance (MCM) complex and modulates the activity of the Dbf4-dependent Cdc7 kinase (DDK). Subsequently, DDK phosphorylates the MCM2–7 helicase, which promotes the activation of dormant origins and ensures the completion of DNA replication (32, 33). Consequently, loss of FANCI function results in fewer activated replication origins, increased inter-origin distance, and impaired cellular proliferation (33).

### 3.2 FANCI installed replication fork protection and restart

Replication stress, induced by factors such as DNA damage or nucleotide depletion (e.g., by hydroxyurea), can cause fork stalling (2). Unprotected forks undergo nucleolytic degradation, inducing genomic instability. The ID2 complex cooperates with RAD51 to protect stalled forks by stabilizing RAD51 filaments, thereby shielding nascent DNA from FAN1-mediated degradation—this depends on FANCI’s DNA-binding activity. *In vitro* data show RAD51 filament stabilization can bypass FANCD2 deletion-induced homologous recombination defects (34). Additionally, FANCD2 collaborates with BRCA2/RAD51 to prevent MRE11-mediated fork degradation (35, 36).

The ID2 complex also facilitates fork restart through the BLM complex (BLMcx: BLM, TOP3α, RMI1/2, RPA). FANCD2 maintains BLM stability, promotes BLMcx assembly, and regulates its activity under stress (37). The ID2 complex exhibits regulatory antagonism: while FANCD2 promotes fork restart, it suppresses dormant origin activation, a function counteracted by FANCI (38). Notably, ATR-mediated phosphorylation of FANCI triggers a functional switch—from activating dormant origins to promoting fork restart and inhibiting origin activation, mimicking FANCD2 (33), suggesting that FANCI possesses a tunable functionality that enables dynamic adaptation to replication stress. Collectively, these findings illustrate that the ID2 complex orchestrates fork protection, fork restart, and the coordination of dormant origin firing in a context-dependent manner to precisely counteract replication stress thus safeguards genome integrity (38).

### 3.3 Mechanisms of FANCI participation in DNA damage repair

Replication stress-induced interstrand crosslinks (ICLs) that block replication and transcription trigger the activation of the FA pathway, with a crucial initial step being the formation of the stable FANCI-FANCD2 (ID2) complex (5).

The ID2 complex facilitates ICL repair through a mechanism of open-closed-locked conformational cycling (39–43). In its open state, the complex functions as a sliding clamp, diffusing freely along duplex DNA. Upon encountering a ss/ds DNA junction, the complex stalls: FANCI binds DNA via its C-arch structure, while FANCD2’s KR helix recognizes the junction. The straightened DNA, along with low-charge adjacent ssDNA, stabilizes the arrested complex (8). Subsequently, DNA binding and ATR-mediated FANCI phosphorylation drive transition to the closed state, exposing FANCD2’s monoubiquitination site, which activates the FA pathway (39, 41, 43). Ubiquitin at the FANCI-FANCD2 interface acts as a “molecular pin”, locking the complex onto DNA, and preventing reopening or dissociation until deubiquitination occurs (43). The locked ID2 complex recruits downstream repair factors (e.g., FAN1, MRE11) to enable nucleolytic incision and ICL repair (44). After the resolution, deubiquitination and dephosphorylation dissociate the complex from DNA, resetting it to the open state, and completing the repair cycle (8).

### 3.4 FANCI’s role in cell fate decision after DNA damage

**Figure 2:**
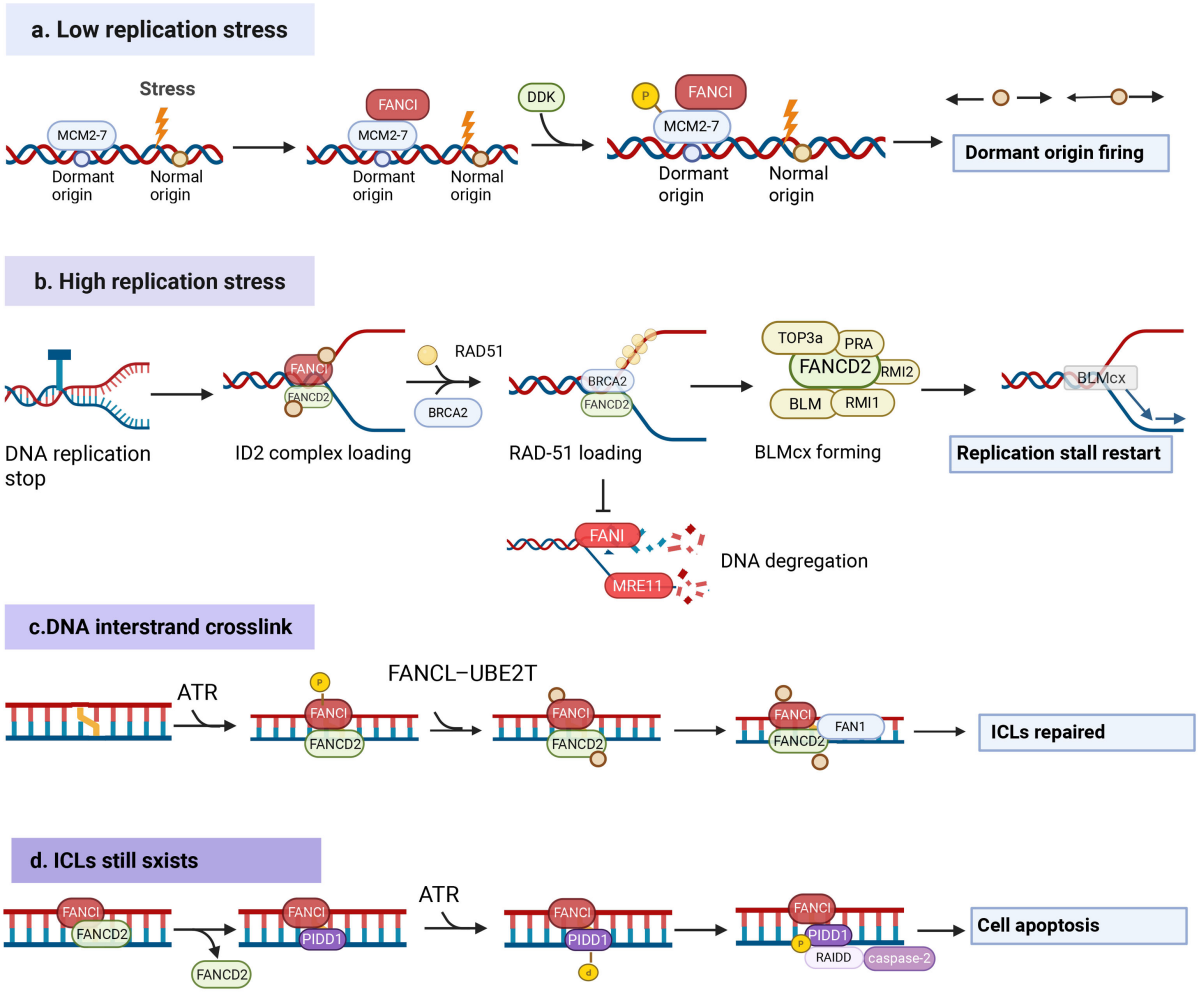
Molecular mechanisms of FANCI in DNA replication and damage repair. **(a)** Dormant origin activation: Under mild replication stress, non-phosphorylated FANCI is recruited to dormant origins and facilitates DDK-mediated phosphorylation of the MCM2–7 complex, leading to origin firing. **(b)** Fork protection and restart: Following replication fork stalling, the ID2 complex is loaded and, alongside BRCA2, recruits RAD51 to protect nascent DNA from degradation by nucleases (e.g., FAN1, MRE11). FANCD2 then promotes fork restart by recruiting the BLM complex (BLMcx). **(c)** ICL repair: The ID2 complex is activated at ICL sites through ATR-mediated phosphorylation and FANCL-UBE2T–mediated monoubiquitination, enabling the recruitment of downstream effectors (e.g., FAN1) for crosslink resolution. **(d)** Cell fate decision: Under irreparable damage, FANCI switches from its repair function to promote assembly of the PIDDosome complex, triggering apoptosis. TOP3α, DNA Topoisomerase III Alpha; RPA, Replication Protein A; RMI1, RecQ-Mediated Genome Instability 1; RMI2, RecQ-Mediated Genome Instability 2. (Created with biorender.com).

Beyond its canonical role in the ID2 complex during ICL repair, FANCI independently regulates critical cell fate decisions. When repair fails or damage is severe, FANCI switches from the FANCD2-dependent repair pathway to promote assembly of the PIDDosome complex (comprising PIDD1, RAIDD, and caspase-2), thereby initiating apoptosis. This functional switch can be triggered by several conditions, including the loss of key downstream nucleases in the FA pathway, excessive ICL accumulation, or aberrant entry of damaged cells into mitosis. Conversely, if PIDDosome assembly fails, FANCI can revert to the FANCD2-dependent repair pathway, demonstrating the plasticity of this cell fate decision (45). This finely tuned regulatory mechanism establishes FANCI as a central molecular decision-maker, critically balancing the restoration of genome integrity against the timely elimination of irreparably damaged cells (**Figure 2**).

As a central guardian of genome stability, a deeper understanding of how FANCI protects replication forks and precisely orchestrates the switch between repair and apoptosis will elucidate the fundamental molecular logic governing cellular homeostasis under replication stress, highlighting its significant research and therapeutic potential.

## 4 FANCI participate in ribosome biogenesis, meiosis, and mRNA export regulation

FANCI exerts critical non-canonical functions beyond DNA repair, including ribosome biogenesis, meiosis, and mRNA export regulation.

### 4.1 Ribosome biogenesis and meiosis

In ribosome biogenesis, deubiquitinated FANCI localizes to the nucleolus (via USP1/USP36) to bind RNA polymerase I and promote rDNA transcription, and interacts with PES1 to facilitate LSU pre-rRNA processing. FANCI participate in ribosome biogenesis when DNA is stable; upon damage, it relocates to the nucleoplasm for repair (22). In meiosis, FANCI is expressed in spermatocytes, sperm, and oocytes; it colocalizes with RPA and interacts with RAD51 to promote D-loop formation, a critical step in homologous recombination (46). Besides, FANCI significantly stimulates FANCD2-mediated nucleosome assembly (47).

### 4.2 mRNA export

**Figure 3:**
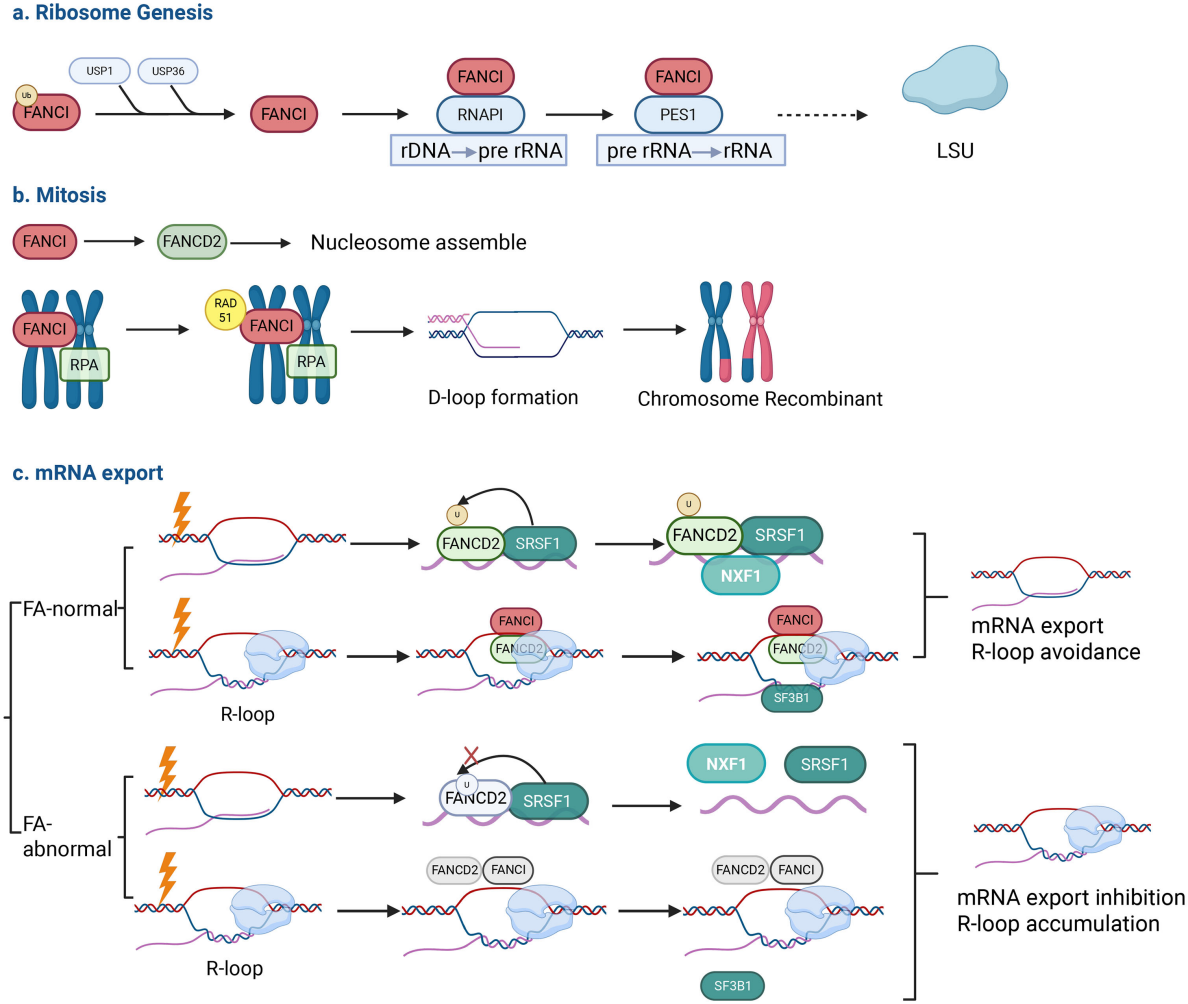
The role of FANCI in ribosome biogenesis, meiosis, and mRNA export regulation. **(a)** Ribosome biogenesis: Deubiquitinated FANCI binds RNAPI to promote rDNA transcription into pre-rRNA, and interacts with PES1 to facilitate pre-rRNA processing, thus driving large ribosomal subunit (LSU) biogenesis. **(b)** Mitosis: FANCI enhances FANCD2-mediated nucleosome assembly; it also associates with RPA to promote RAD51-dependent D-loop formation and meiotic homologous recombination. **(c)** mRNA export and R-loop resolution: In functional FA pathways, SRSF1 induces RNA-dependent FANCD2 monoubiquitination to promote SRSF1-NXF1 complex assembly, facilitating mRNA export and R-loop resolution. The ID2 complex further suppresses R-loop accumulation via interaction with SF3B1. Conversely, FA pathway defects impair FANCD2 monoubiquitination and ID2 complex formation, leading to defective mRNA export and pathological R-loop accumulation. USP, ubiquitin specific peptidase. (Created with biorender.com).

In mRNA export regulation, FANCI and FANCD2 interact with SF3B1 to modulate splicing factor dynamics. Under replication stress, FANCI promotes SF3B1 release from nuclear speckles to suppress R-loop accumulation and resolve transcription-replication conflicts (48). Additionally, SRSF1 induces RNA-dependent FANCD2 monoubiquitination to facilitate SRSF1-NXF1 complex assembly and mRNA export (24). The ID2 complex binds ssRNA/DNA to inhibit pathogenic R-loop formation and stimulate its own monoubiquitination for FA pathway activation (49) (**Figure 3**). So essentially, FANCI coordinates transcription and replication by regulating RNA transcription and splicing, thereby preventing deleterious interference between these processes.

As a multifunctional protein, FANCI coordinates diverse core cellular processes. While its roles in DNA repair, transcription, and meiosis are well-defined, how FANCI binds to RNA polymerase I and participates in pre-rRNA processing remain to be further investigated. Potential research directions include resolving R-loops formed during RNAPI transcription (50) and investigating whether FANCI coordinates replication bubbles within rDNA repeats (51). Besides, FANCI’s precise function in pre-rRNA processing awaits reconstitution with purified components beyond PES1.Elucidating these novel mechanisms is essential for understanding FANCI’s role in human diseases.

## 5 Impact of FANCI gene mutations on its function

Mutations in the FANCI gene impair its function through multiple mechanisms, ultimately leading to DNA repair deficiency and the development of associated diseases. Common mechanisms include the generation of premature termination codons (PTCs) that trigger nonsense-mediated mRNA decay (NMD) (11), production of full-length but unstable proteins prone to degradation, or disruption of the start codon (52). Pathogenic mutations often disrupt key functional domains, compromising DNA binding (11, 53), nuclear localization (11, 28), or interaction with FANCD2, thereby blocking the formation of the ID2 complex (11). Additionally, some mutations impair downstream FANCD2 monoubiquitination (54, 55), attenuating FA pathway activation and contributing to diseases such as premature ovarian insufficiency (POI) (56), malignant melanoma (57) and ovarian cancer. Specific mutations and their functional alterations refer to **Table 1** (10, 11, 54–57).

**Table 1:** FANCI mutation sites and altered protein functions.

Mutation site	Amino acid change	Functional alterations	Disease	References
NR	p.Met1?	Start codon mutation; full-length protein loss	FA with VACTERL-H Association	(9, 52)
NR	p.E1117fs	Frame shift; abnormal protein function	FA	(52, 58)
NR	p.H858_R879del	Key protein region deletion	FA with VACTERL-H Association	(9, 52)
NR	p.R1299*	Impaired nuclear localization	FA with VACTERL-H Association	(52)
NR	p.R614*	Truncated protein	FA with VACTERL-H Association	(58)
c.3899_3900insG	p.D1301Gfs*3	Impaired nuclear localization	FA with VACTERL-H Association	(11, 58)
c.3799_3802delCTTT	p.S1268Rfs*5	Impaired nuclear localization	FA with VACTERL-H Association	(11)
c.1461T>A	p.Y487*	NMD	FA with VACTERL-H Association	(11)
c.3041G>A	p.C1014Y	Decreased FANCD2 interaction ability	FA with VACTERL-H Association	(11)
c.1039T>C	p.S347P	Decreased DNA-binding ability	FA with VACTERL-H Association	(11)
c.1201_1201delG	p.G401Efs*3	NMD	FA with VACTERL-H Association	(11)
c.97C>T	p.L33F	Significantly reduced FANCD2 ubiquitination	POI	(56)
c.1865C>T	p.S622L	Significantly reduced FANCD2 ubiquitination	POI	(56)
c.959A>G	p.Q320R	Significantly reduced FANCD2 ubiquitination	POI	(56)
c.158-2A>G	p.S54Pfs*5	Truncated protein	POI, FA with VACTERL-H Association	(10, 56)
c.3006 + 3A>G	abnormal splicing	In-frame deletion	FA	(59)
c.3442_3443del	p.L1148fs	Truncated protein	FA	(59)
c.1813C>T	p.L605F	Significantly reduced FANCD2 ubiquitination	Ovarian cancer	(55)
c.3896G>T	p.R1299L	No effect on known functional domains	Dysplastic nevus	(57)
c.3111_3123del	p.S1038Lfs*19	Affects solenoid 4 domain; Decreased FANCD2 interaction ability	Melanoma	(57)
c.2768A>G	p.Y923C	Affects solenoid 3 domain	Melanoma	(57)
NR	p.R1285Q	Decreased DNA-binding ability; inhibits FANCD2 ubiquitination	NR	(54)
NR	p.K294E	Decreased DNA-binding ability; inhibits FANCD2 ubiquitination	NR	(54)
NR	p.K339E	Decreased DNA-binding ability; inhibits FANCD2 ubiquitination	NR	(54)

FA, Fanconi Anemia; VACTERL-H, at least three of the following: vertebral anomalies, anal atresia, cardiac anomalies, tracheoesophageal fistula, esophageal atresia, renal anomalies, limb anomalies, and hydrocephalus; POI, premature ovarian insufficiency; NMD, nonsense-mediated decay; *, premature stop codon; NR, not reported.

Notably, FANCI’s role in cancer susceptibility remains uncertain: while one study indicated its mutations do not affect breast cancer risk (60), another reported the c.1813C>T mutation is enriched in breast cancer (55). To fully leverage FANCI for early cancer diagnosis and risk stratification, future studies should define mutation-specific molecular outcomes, distinguish germline from somatic origins, and clarify its context-dependent tumorigenic roles.

## 6 FANCI promotes the onset and progression of malignant tumors

### 6.1 FANCI promotes tumorigenesis by affecting genome stability

Across multiple malignancies, FANCI exerts oncogenic effects linked to key cellular processes and genomic stability (61). In breast cancer, high FANCI expression correlates with poor prognosis, increased proliferation, and enhanced migration. Mechanistically, it interacts with PARP1 to promote its nuclear localization and PARylation activity, strengthening DNA damage repair and tumor cell survival under genotoxic stress (62, 63). Upstream, CTD phosphatase 1 regulates FANCI chromatin loading, facilitating its association with γ-H2AX and SQ domain phosphorylation to boost FANCA/FANCD2 focus formation, homologous recombination efficiency, and breast cancer cell growth (64). FANCI also modulates splicing, DNA replication, and cell cycle progression in cervical cancer via cancer-associated kinases and E2F factors (17); and link to cell cycle regulation, VEGF signaling, immunity, and ribonucleoprotein biogenesis in HCC (18).

In lung adenocarcinoma, FANCI knockout causes a genome-wide reduction of R-loop signals, particularly at the promoter regions of genes involved in the Ras/MAPK signaling pathway. This reduction blocks their active transcription, thereby suppressing tumorigenesis through decreased proliferation, invasion and enhanced apoptosis (23). Notably, in normal cells, the ID2 complex suppresses pathogenic R-loop formation by binding ssRNA or ssDNA and activating FA pathway, its loss would increase R-loops (49). However in cancer cells, due to oncogenic reprogramming, FANCI promotes R-loop formation at the promoter regions of oncogenes and activates their transcription, thereby enhancing oncogene expression (23). It suggests that FANCI exhibits opposite orientations of R-loop regulation between normal and cancerous contexts.

As a core genome stability regulator, FANCI enhances DNA repair in diverse tumors.Currently, the mechanisms by which FANCI promotes R-loop formation in cancer cells and the precise binding sites of FANCI on R-loops remain unclear. Addressing these questions through multi-omic and multi-model analyses will be essential to assess the pan-cancer therapeutic potential.

### 6.2 FANCI promotes tumorigenesis by regulating key signaling pathways

#### 6.2.1 PI3K/AKT pathway

PI3K/AKT signaling is a highly conserved pathway in eukaryotic cells, and its dysregulation—such as PI3K hyperactivation, PTEN loss, and aberrant Akt activation—plays a critical role in tumor progression and therapeutic resistance (65). Emerging evidence indicates that FANCI is involved in the regulation of AKT signaling. Yin et al. found FANCI knockdown in hepatocellular carcinoma (HCC) downregulated p-AKT and Cyclin D1 while upregulating GSK-3β (66), indicating that FANCI promotes Akt activation and contributes to hepatocellular carcinoma progression. Li et al. reported FANCI deficiency in glioma reduced p-AKT and Bcl-2 while increasing Bax,suggesting that FANCI promotes cell survival and malignant progression through the Akt–Bcl-2 axis (16).

On the other hand, Zhang et al. reported that FANCI acts as a negative regulator of Akt activation in mammary epithelial cells. Loss of FANCI reduced the interaction between PHLPP1 and Akt, thereby enhancing Akt phosphorylation and suppressing DNA damage-induced apoptosis (66). Collectively, FANCI regulates tumor cell proliferation and survival by modulating the Akt signaling pathway. However, its precise action may vary with cell type and biological context.

#### 6.2.2 CHK1/2-P53-P21 pathway

Checkpoint kinase 1 (CHK1) and CHK2 are core regulators of the DNA damage response (60). Typically inactive, they are phosphorylated and activated by ATR (CHK1) and ATM (CHK2) upon genotoxic stress. A key downstream target is the tumor suppressor p53; CHK1/2-mediated phosphorylation stabilizes p53 and promotes transcription of the cell-cycle inhibitor p21, ultimately inducing cell-cycle arrest or apoptosis (67). In ovarian and prostate cancers, FANCI silencing exacerbates DNA damage and consequently hyperactivates the CHK1/2–p53–p21 signaling axis. This leads to p21 upregulation, triggering cell-cycle arrest and apoptosis (19). The cellular consequences of FANCI loss exhibit p53 dependency. In prostate cancer models, FANCI deletion induces G1-phase arrest, suppresses proliferation, and enhances carboplatin sensitivity exclusively in p53 wild-type cells. These effects are absent in p53-deficient or p53-mutated cells (20).

#### 6.2.3 MEK/ERK/MMPs pathway

**Figure 4:**
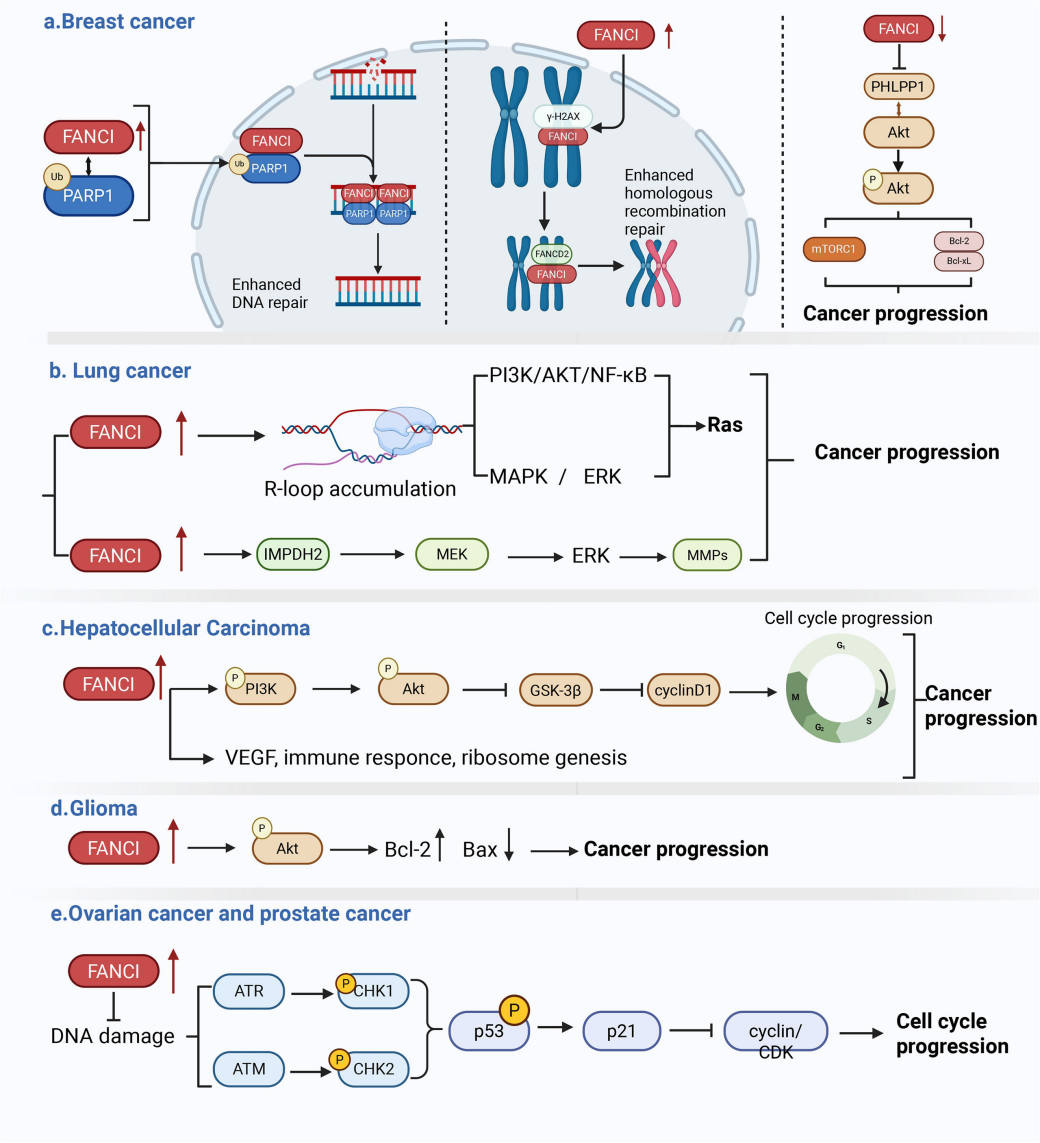
FANCI promotes tumorigenesis and progression by affecting genomic stability and signaling pathways. **(a)** Breast cancer: FANCI overexpression enhances DNA repair via PARP1 interaction and homologous recombination (γ-H2AX binding, FANCA/FANCD2 focus formation); FANCI loss may promote tumorigenesis through Akt pathway dysregulation. **(b)** Lung cancer: FANCI drives proliferation via R-loop accumulation and PI3K/AKT/NF-κB/MAPK/ERK activation, and invasion through the IMPDH2–MEK/ERK–MMPs axis. **(c)** Hepatocellular carcinoma: FANCI activates PI3K/Akt signaling to promote cell cycle progression (GSK-3β/cyclin D1) and participates in VEGF signaling and ribosome biogenesis. **(d)** Glioma: FANCI activates Akt to upregulate Bcl-2 and downregulate Bax, inhibiting apoptosis and enhancing tumor survival. **(e)** Ovarian & prostate cancers: FANCI modulates the CHK1/2–p53–p21 pathway to regulate cell cycle progression and therapeutic sensitivity in response to DNA damage. PARP1, poly(ADP-ribose) polymerase 1; γ-H2AX, phosphorylated H2A histone family member X (Ser139); Akt, protein kinase B; PI3K, phosphoinositide 3-kinase; NF-κB, nuclear factor kappa-light-chain-enhancer of activated B cells; MAPK, mitogen-activated protein kinase; ERK, extracellular signal-regulated kinase; MEK, MAPK/ERK kinase; MMPs, matrix metalloproteinases; IMPDH2, inosine monophosphate dehydrogenase 2; GSK-3β, glycogen synthase kinase 3 beta; VEGF, vascular endothelial growth factor; Bcl-2, B-cell lymphoma 2; Bax, Bcl-2-associated X protein; CHK1/2, checkpoint kinase 1/2; p53, tumor protein p53; p21, cyclin-dependent kinase inhibitor 1. (Created with biorender.com).

In lung adenocarcinoma (LUAD), FANCI promotes tumor progression by stabilizing IMPDH2 and activating the MEK/ERK signaling pathway, forming a FANCI–IMPDH2–MEK/ERK–MMPs axis that drives cell migration and invasion (21). Notably, recent studies in triple-negative breast cancer (TNBC) have identified a novel prieurianin-type limonoid, DHL-11, which exerts potent antitumor effects by directly binding to the non-catalytic pocket of IMPDH2 and disrupting its interaction with FANCI, thereby inducing IMPDH2 degradation. This leads to impaired guanine synthesis, increased ROS accumulation, and enhanced DNA damage, ultimately suppressing tumor growth, metastasis, and patient-derived organoid viability with favorable biosafety profiles (68). Collectively, these findings further highlight the critical role of the FANCI–IMPDH2 axis and MEK/ERK signaling pathway in tumor progression and underscore its potential as a promising therapeutic target. (**Figure 4**).

### 6.3 FANCI promotes tumorigenesis by altering the tumor immune microenvironment

**Figure 5:**
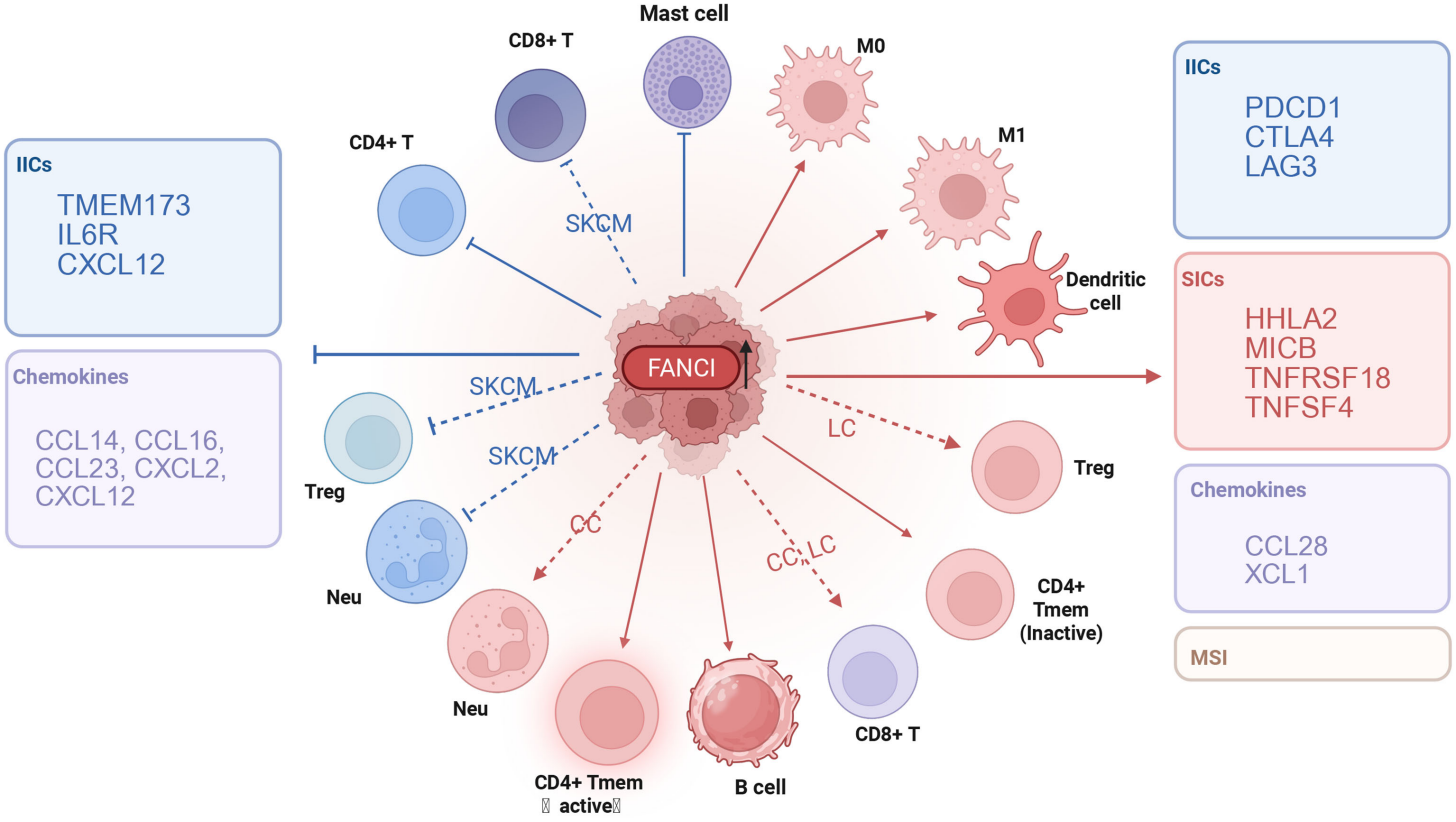
FANCI promotes tumor development by altering the tumor immune microenvironment. Immune cell regulation: FANCI upregulation modulates immune cell infiltration in a cancer-type-specific manner, commonly upregulating macrophages (M0/M1), dendritic cells, B cells, and memory CD4^+^ T cells (Tmem), while downregulating CD4^+^ T cells and mast cells. Tregs, CD8^+^ T cells, and neutrophils show opposing regulatory patterns in LC/CC vs. SKCM. Immune checkpoints & chemokines: FANCI expression correlates positively with checkpoints (PD-1, CTLA-4, LAG-3, HHLA2) and negatively with others (TMEM173/STING, IL6R); it also differentially regulates chemokines, upregulating CCL28/XCL1 and downregulating CCL14/16/23 and CXCL2/12. Microsatellite instability: FANCI expression positively correlates with MSI scores. PD-1, programmed cell death protein 1; CTLA-4, cytotoxic T-lymphocyte-associated protein 4; LAG-3, lymphocyte-activation gene 3; HHLA2, HERV-H LTR-associating protein 2; TMEM173/STING, transmembrane protein 173; IL6R, interleukin-6 receptor; CXCL, C-X-C motif chemokine ligand; CCL, C-C motif chemokine ligand; Tmem, memory T cell; Treg, regulatory T cell; LC, lung carcinoma; CC, colon carcinoma; SKCM, skin cutaneous melanoma; MSI, microsatellite instability. (Created with biorender.com).

FANCI overexpression closely correlates with tumor immune microenvironment (TIME) reprogramming, and its immunomodulatory effects are cancer-type specific. In skin cutaneous melanoma (SKCM), FANCI expression negatively correlates with immunosuppressive Tregs and cytotoxic CD8^+^ T cells but positively with pro-inflammatory M1 macrophages, suggesting a dual role in driving inflammation and impairing anti-tumor immunity (69). High FANCI expression suppresses M1 macrophage activation in lung adenocarcinoma (LUAD) to facilitate immune evasion (70), whereas it correlates positively with multiple immune cell infiltrates in cervical cancer (17). In hepatocellular carcinoma (HCC), FANCI promotes an immunosuppressive TIME by enhancing CD8^+^ T cell infiltration alongside immunosuppressive components (Tregs, Th2 cells, M2 macrophages) (18). In HCC, FANCI expression is also linked to immune checkpoints, chemokines, and microsatellite instability (MSI): it correlates positively with both inhibitory (PD-1, CTLA-4, LAG-3) and stimulatory (HHLA2, MICB) checkpoints, differentially regulates chemokine expression, and associates with elevated MSI scores (18) (**Figure 5**). Overall, FANCI is a key regulator of TIME, and clarifying its cancer-specific immunomodulatory mechanisms is critical for developing FANCI-targeted immunotherapies.

## 7 Upstream regulatory factors affecting FANCI function

FANCI expression and functional activity are tightly regulated by a multifaceted upstream network, primarily via post-translational modifications (phosphorylation, monoubiquitination, deubiquitination) and non-coding RNA-mediated regulation.

### 7.1 Effects of phosphorylation and dephosphorylation on FANCI function

Phosphorylation of FANCI serves as a central switch controlling activation of the FA pathway. It is widely accepted that ATR kinase-mediated phosphorylation licenses downstream monoubiquitination of the ID2 complex and its recruitment to nuclear foci (71, 72), by triggering a conformational change in FANCD2 from an open to a closed state and exposing ubiquitin acceptor sites to facilitating ubiquitination (8, 39). While it is widely recognized that Ser556 phosphorylation licenses ubiquitination, recent studies have shown that Ser559/565 phosphorylation occurs downstream of ubiquitination and functions to inhibit FANCD2 deubiquitination, thereby stabilizing the ID2 complex and prolonging the repair process (72, 73). Thus, ATR kinase promotes the accumulation of ID2 in its ubiquitinated, DNA repair-competent form.

Besides prolonging ubiquination, FANCI phosphorylation also enables context-specific responses to replication stress (74). Under low stress, phosphorylation of FANCD2 prevents ID2 from loading onto chromatin, thereby keeping the FA pathway in a suppressed state (75). In contrast, severe stress triggers ATR-mediated phosphorylation, which inhibits the activation of dormant replication origins and promotes fork restart (33, 76). It is worth noting that Diyang et al. showed that under DNA damage, PP2A dephosphorylates an inhibitory cluster in FANCD2 to license its loading for repair initiation, thus initiating the FA pathway (77).

Overall, the function of the ID2 complex is regulated by a complex phosphorylation network, whereas the precise timing and regulation of ID2 dephosphorylation remain unclear, and further investigation is required.

### 7.2 Effects of ubiquitination and deubiquitinition on FANCI function

Monoubiquitination of the ID2 complex is a critical step in ICL repair. There is a general consensus that monoubiquitination of the ID2 complex serves as the central locking event, specifically FANCI at Lys523, which traps the complex on DNA and recruits downstream nucleases for ICL excision (40, 43, 78). However, there is controversy regarding how the FANCI-FANCD2 complex reaches and stalls at the damage site. Conventional studies showed after DNA crosslink damage, BRCA1 guides FANCI to bring the repair team to the site (79). RAD18 facilitates ID2 loading and monoubiquitination during S phase via direct binding to FANCD2 (80). However, recent studies indicate that FANCI directly recognizes ss-dsDNA junctions via its unique structure, potentially serving as the initial step in damage perception (8). The ubiquitinated ID2 complex then assembles into chromatin filaments to recruit downstream effectors such as FAN1 for ICL repair (44, 81–83). FANCI monoubiquitination is catalyzed by the FANCL–UBE2T module of the FA core complex (52, 58, 78, 82, 84, 85). Defined DNA structures, ssRNA, and R-loops strongly stimulate FANCD2 monoubiquitination, while unstructured ssDNA or chromatinized DNA do not (49, 53, 86).

As for deubiquitinition, USP1–UAF1 deubiquitinates FANCI before repair and FANCD2 after repair, cycling ID2 between active and reset states (87). Notably, USP1 depletion increased ID2 monoubiquitination but impaired ubiquitination-dependent FANCI phosphorylation, revealing that timely FANCD2 deubiquitination is required for FA pathway function (72). The ubiquitination–deubiquitination dynamics of FANCD2 and FANCI are temporally regulated (52, 87), as FANCI ubiquitination stabilizes FANCD2-Ub to resist deubiquitination and prolong ID2 residence on DNA (41, 82, 86).

To sum up, phosphorylation triggers a conformational change, enabling ubiquitination to lock the ID2 clamp on DNA, followed by deubiquitination that resets the complex for another repair cycle. Future studies should address how these post-translational modifications are coordinated in time and space to ensure faithful ICL repair.

### 7.3 Effects of non-coding RNAs on FANCI function

MicroRNAs (miRNAs) post-transcriptionally regulate FANCI expression. For example, miR-218 targets the 3’ untranslated region of FANCI mRNA, promoting its degradation and suppressing protein synthesis. In LUAD, downregulation of miR-218 elevates FANCI levels, driving metastasis and therapy resistance (88). Alternative splicing of FANCI pre-mRNA, regulated by RNA-binding proteins, generates distinct isoforms like FANCI-12 and FANCI-13. FANCI-12 confers radioresistance, while FANCI-13 does not. Inhibition of the splicing factor PRP3 prevents FANCI-12 production, disrupts splicing, and enhances radiosensitivity (89).

**Figure 6:**
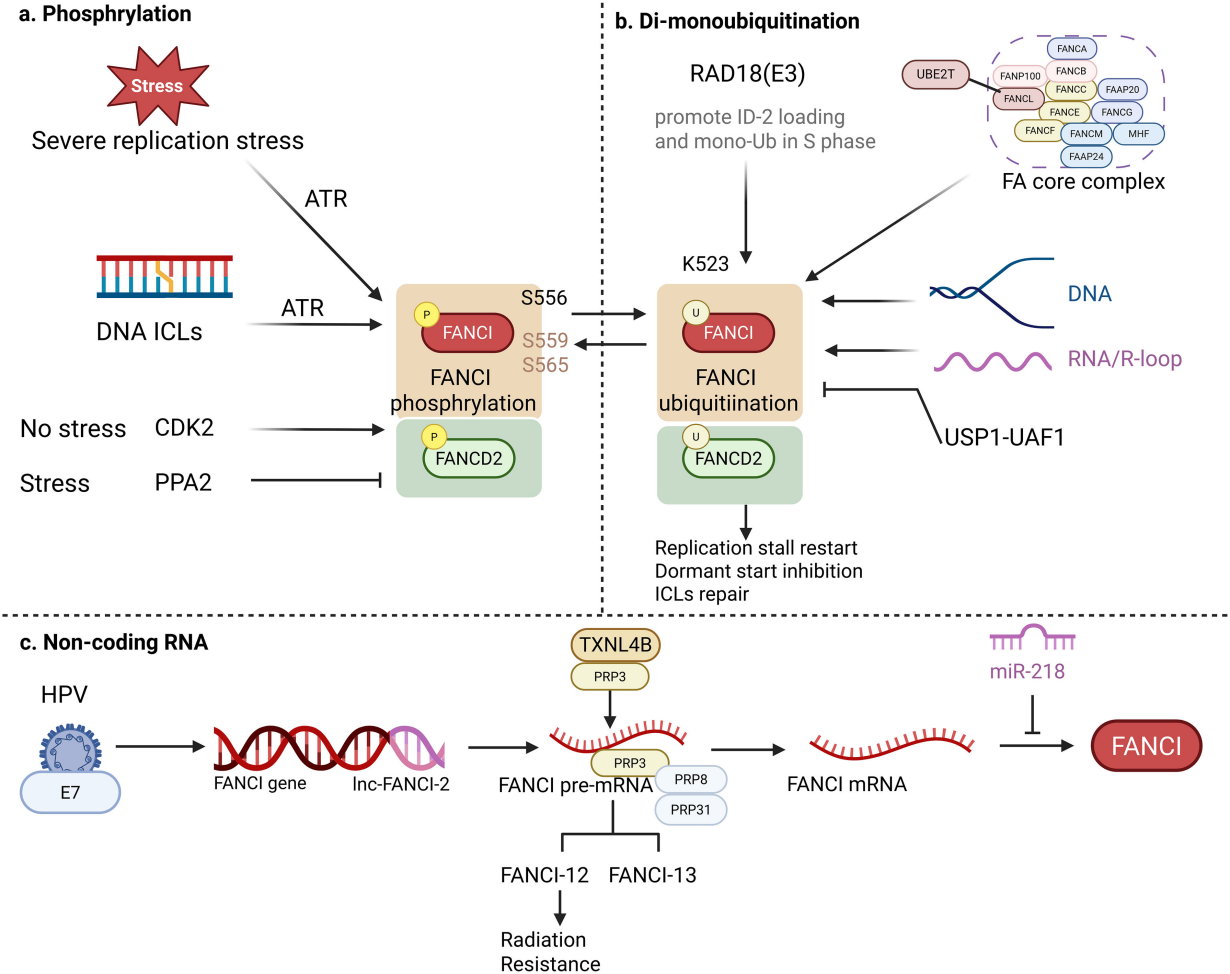
Upstream regulatory factors affecting FANCI function. **(a)** Phosphorylation/dephosphorylation: Under replication stress or ICLs, ATR phosphorylates FANCI (e.g., Ser556, Ser559, Ser565) to promote ubiquitination and repair. Conversely, CDK2-mediated FANCD2 phosphorylation under basal conditions is reversed by PP2A upon DNA damage to initiate repair. **(b)** Ubiquitination/deubiquitination: The FANCL–UBE2T complex, aided by the FA core complex, monoubiquitinates FANCI at Lys523. This is enhanced by nucleic acids (DNA, R-loops) and the E3 ligase RAD18. USP1–UAF1 mediates deubiquitination to reset the ID2 complex. **(c)** Non-coding RNA regulation: 1) HPV E7 upregulates lnc-FANCI-2 and FANCI transcription. 2) TXNL4B mediates PRP3 nuclear import, enabling the PRP3/PRP8/PRP31 spliceosome to generate the radioresistance-conferring FANCI-12 isoform. 3) miR-218 targets FANCI mRNA for degradation; its loss in LUAD leads to FANCI overexpression. (Created with biorender.com).

The long non-coding RNA lnc-FANCI-2, located downstream of FANCI, interacts with FANCI in cervical cancer. HPV oncoprotein E7 upregulates both lnc-FANCI-2 and FANCI, activating ATM/ATR kinases, promoting FANCI phosphorylation, and enhancing DNA repair (90) (**Figure 6**).

Targeting regulatory networks of non-coding RNAs offers potential for reversing chemoresistance and radioresistance in FANCI-driven cancers. Understanding the complex interactions within this network is crucial for developing targeted therapeutic strategies.

## 8 FANCI and disease diagnosis, treatment, and prognosis

### 8.1 FANCI as a biomarker for disease diagnosis and risk prediction

Pathogenic FANCI mutations typically follow an autosomal recessive inheritance pattern. The majority of affected individuals are compound heterozygotes, inheriting distinct pathogenic alleles from each parent. Examples include the p.Leu33Phe/p.Ser622Leu and p.Ser54Profs*5/p.Gln320Arg genotypes identified in primary ovarian insufficiency (POI) (56), and the c.3006 + 3A>G/p.Leu1148fs mutation in Fanconi anemia (FA) patients (59). Less frequently, homozygous mutations—such as the p.Leu605Phe variant identified in both ovarian cancer and FA (55)—have been reported. Furthermore, loss of heterozygosity (LOH) at the FANCI locus has been detected in tumor specimens, supporting a potential “second-hit” mechanism in FANCI-associated tumorigenesis (57).

FANCI is significantly upregulated in the majority of tumor types, including SKCM (69), cervical cancer (17), LIHC (18), prostate cancer (20), LUAD (21), and colon adenocarcinoma and pancreatic adenocarcinoma (14). In LIHC, FANCI expression positively links to tumor grade, stage and hepatitis B history, and negatively correlates with promoter methylation; it also associates with immune cell infiltration and tumor mutation burden in some cancers (18). Genomic alterations of FA pathway genes are common in prostate adenocarcinoma (20). In ovarian cancer, low FANCI expression correlates with FIGO staging differences in HGSOC and non-HGSOC, while high expression does not, implying its role as a marker for lower FIGO stage and high-grade histology (91). Collectively, FANCI serves as a promising diagnostic biomarker with therapeutic relevance.

### 8.2 FANCI and personalized precision therapy

#### 8.2.1 FANCI as a direct therapeutic target

Accumulating evidence demonstrates that FANCI knockdown directly suppresses tumorigenesis by inhibiting cancer cell proliferation, migration, and invasion, while inducing apoptosis and cell cycle arrest across multiple cancer types, including melanoma, NSCLC, LIHC, and glioma (16, 21, 69, 70, 92, 93). These anti-tumor effects are consistently recapitulated in animal models, where FANCI depletion significantly impedes tumor growth, validating its potential as a potential therapeutic target (16, 70). Upstream regulators of FANCI represent alternative therapeutic targets. Targeting the E2 enzyme UBE2T in NSCLC disrupts FANCI monoubiquitination and reverses its oncogenic activity, validating this indirect strategy (92). Targeting critical upstream regulators may offer a favorable therapeutic window by modulating pathway activity with reduced risk of systemic toxicity. Elucidating its mechanisms in human reproduction is of great significance for the rational design of therapeutic regimens.

#### 8.2.2 FANCI as a sensitization target for tumor therapy

As a key therapeutic sensitizer, targeting FANCI represents a promising strategy to reverse chemoresistance. In ovarian and prostate cancers, FANCI knockdown exacerbates DNA damage and sensitizes cells to platinum-based agents like carboplatin (19), potentially converting therapy-resistant populations into vulnerable ones (20). Consequently, combining FANCI inhibitors with DNA crosslinking chemotherapeutics (e.g., cisplatin, carboplatin) may allow for dose reduction to mitigate toxicity while enhancing therapeutic efficacy. FANCI inhibition also extend the utility of PARP inhibitors beyond BRCA-mutant cancers. Evidence shows that FANCI depletion induces PARP1 chromatin retention, thereby potentiating the efficacy of Talazoparib in breast cancer models, including those with intact homologous recombination (63). Radiosensitization represents another therapeutic opportunity. Inhibition of PRP3, which mediates TXNL4B-dependent splicing of FANCI pre-mRNA, suppresses the production of pro-survival FANCI -12, thereby sensitizing lung cancer cells to radiotherapy (89). FANCI expression correlates with immunotherapy response: lower levels associate with better outcomes, accompanied by enhanced immune checkpoint expression, MHC presentation, and stable antigen processing (69). Conversely, In LIHC, high FANCI links CD8^+^ T cell infiltration and upregulated inhibitory checkpoints, supporting combination therapy of FANCI inhibition plus immune checkpoint blockade (18).

The core therapeutic value of FANCI targeting is its role as a potent “therapeutic sensitizer.” Exploring FANCI inhibitors in rational combinations is a promising oncology frontier, offering to overcome drug resistance, expand eligible patients, and establish novel regimens with improved efficacy and reduced toxicity.

#### 8.2.3 Target-related risks and translational prospects

Given the central role of FANCI in DNA repair, potential on-target toxicities must be carefully evaluated before utilizing in clinical case. Animal experiments demonstrate that FANCI’ s knockdown link to reproduction disorders, including embryonic lethality, male infertility and female POI (25, 94). To address reproductive toxicity, the PCN-222-Mn nanozyme crosses the blood-testis barrier, scavenges ROS, and enhances autophagy (95), while co-delivery micelles package chemotherapeutics with ovarian protectants (96). Combination regimens also hold promise. The optimal ratio of palbociclib (5 µM) to talazoparib (500 nM) achieves strong synergy (CI < 1), potentially minimizing toxicity via lower individual doses (63). Notably, preclinical studies have demonstrated that SKY-1214, which degrades FANCI and FANCL mRNAs via splicing modulation, exhibits potent activity in multiple myeloma and lymphoma models (97). However, before clinical application, such strategies must undergo rigorous toxicity assessments, and treated patients require regular follow-up. Although no specific delivery strategy for FANCI has been reported to date, conjugating anti-FANCI small-molecule inhibitors or PROTACs to antibodies targeting tumor-surface antigens thereby enabling endocytosis and intracellular release of the payload represents a promising direction for future research.

### 8.3 The impact of FANCI on prognostic evaluation

Elevated FANCI expression correlates with poor prognosis in multiple cancers (breast, cervical, ovarian, NSCLC, LIHC, ACC, LUAD) (17–19, 21, 63, 70, 92, 93). In SKCM, high FANCI induces immunosuppression and reduces immunotherapy efficacy, while low FANCI improves outcomes (69). Mechanistically, FANCI overexpression promotes LUAD metastasis via ECM and immune modulation (88). FANCI mutation phenotype depends on the affected protein domain and genetic modifiers, providing prognostic value for FA patients. N-terminal deletions cause severe malformations, while C-terminal lesions impair DNA repair. A pediatric case with N-terminal-sparing FANCI mutation showed mild anomalies, but co-inherited ALDH2 risk variant accelerated bone marrow failure, confirming “mutation-site dictates phenotype” and modifier effects (59) (**Table 2**). The clinical significance of FANCI is underscored by its dual role: its frequent overexpression in sporadic cancers and its locus-specific mutations in heritable fanconi anemia collectively establish it as a biomarker of substantial prognostic relevance. These insights provide a robust molecular basis for risk stratification in sporadic cancers and enable more precise prognostic predictions for individuals.

**Table 2:** The role of FANCI in disease diagnosis, treatment and prognosis assessment.

Function	Category	Key findings	Disease type	Clinical significance	References
Biomarker	Altered Expression	FANCI is upregulated in multiple cancers	SKCM, CC, LIHC, PC, LUAD, CRC, PAC, Glioma	Diagnostic biomarker	(14, 16, 17, 19–21, 69, 70, 91–93)
Biomarker	Somatic Genomic Alteration	Somatic FANCI copy number variations exist	OC, PC	Somatic alterations with increased cancer risk	(19, 55)
Biomarker	Germline Genomic Alteration	Germline pathogenic variants of FANCI exist.	FA, PC, Melanoma	Germline variants associated with hereditary cancer risk	(11, 54, 55, 57)
Biomarker	Genetic Disease	Biallelic pathogenic mutations cause FA	FA,VACTERL Association	FANCI is a pathogenic gene for FA; genetic counseling recommended	(11)
Biomarker	TME	FANCI correlates with immune markers	CC, SKCM	Potential immune checkpoint inhibitor responsiveness predictor	(14, 17, 18, 69, 70)
Therapeutic Target	Direct Targeted Therapy	FANCI knockdown inhibits cancer proliferation/invasion	Glioma	FANCI itself is a highly promising direct anti-cancer drug target	(16)
Therapeutic Target	Direct Targeted Therapy	Upstream targeting restores UBE2T and inhibits FANCI function	NSCLC	FANCI upstream factor is a highly promising direct anti-cancer drug target	(92)
Therapeutic Target	Chemotherapy Sensitizer	FANCI impairment impairs ICL repair, increasing platinum sensitivity	OC, PC	FANCI inhibitors are expected to reverse platinum-based chemotherapy resistance	(19, 20, 54)
Therapeutic Target	PARP Inhibitor Sensitizer	FANCI inhibition enhances PARP inhibitor efficacy	BC	FANCI expands PARP inhibitor beneficiaries to BRCA wild-type patients	(63)
Therapeutic Target	Radiotherapy Sensitizer	Targeting TXNL4B increases radiosensitivity	NSCLC	Targeting FANCI splicing network provides a new strategy to overcome radioresistance	(89)
Prognostic Assessment	Poor Prognosis	High FANCI expression correlates with worse overall survival	BC, CC, OC, NSCLC, LIHC, ACC, LUAD	FANCI signaling network provides an strategy to inhibit tumor survival/metastasis	(14, 17–21, 57, 69, 70, 91, 93)
Prognostic Assessment	Genetic Disease Prognosis	Biallelic mutations in FA correlate with VACTERL association	FA, VACTERL Association	Mutation sites are associated with clinical phenotypes	(11)
Safety Concern	Spermatogenesis	FANCI deficiency causes male infertility	FA, NOA	FANCI deficiency affects spermatogenesis, poses challenges to FANCI-targeted therapy	(25)
Safety Concern	Embryonic Development	Biallelic FANCI deficiency causes fetal death and brachygnathia	FA, VACTERL Association	FANCI deficiency affects embryonic development; poses challenges to FANCI-targeted therapy	(94)

SKCM, Skin Cutaneous Melanoma; CC, Cervical Cancer; LIHC, Liver Hepatocellular Carcinoma; PC, Prostate Cancer; LUAD, Lung Adenocarcinoma; CRC, Colorectal Adenocarcinoma; PAC, Pancreatic Adenocarcinoma; OC, Ovarian Cancer; FA, Fanconi Anemia; NSCLC, Non-Small Cell Lung Cancer; BC, Breast cancer; ACC, Adrenocortical Carcinoma; NOA, Non-Obstructive Azoospermia; TME, Tumor Microenvironment; PARP, Poly ADP-Ribose Polymerase; TXNL4B, Thioredoxin Like 4B; UBE2T, Ubiquitin Conjugating Enzyme E2 T.

## 9 Issues and prospects

Despite substantial progress in elucidating the roles of FANCI in DNA damage repair, tumorigenesis, and reproduction, its precise regulatory mechanisms and associated molecular networks in cancer remain to be fully defined. In particular, FANCI-mediated regulation of the Akt signaling pathway suggests a critical role in coordinating tumor cell proliferation and survival, and further clarification of its mechanistic function within this pathway may provide a valuable basis for identifying novel therapeutic targets. Emerging evidence also links FANCI to tumor immunity, indicating its involvement in regulating the tumor microenvironment, including immune cell infiltration, checkpoint signaling, and antigen presentation. Furthermore, FANCI has been reported to interact with IMPDH2, a key enzyme that promotes tumor invasion and metastasis through purine metabolic reprogramming. This suggests that FANCI-mediated DNA damage responses may be closely coupled with metabolic adaptation in cancer cells, although this area requires further investigation.

From a translational perspective, targeting FANCI may enhance tumor sensitivity to DNA-damaging therapies, radiotherapy, and immunotherapy, thereby overcoming treatment resistance. Advances in precision medicine and targeted delivery strategies may further enable selective modulation of FANCI activity in tumors while minimizing toxicity. Overall, FANCI represents a promising therapeutic target that integrates DNA repair, cell cycle regulation, tumor immunity, and metabolic adaptation. Continued investigation of its molecular functions is expected to provide new opportunities for improving cancer therapy and advancing precision oncology.
